# Dietary intake and age at natural menopause: results from the UK Women’s Cohort Study

**DOI:** 10.1136/jech-2017-209887

**Published:** 2018-04-30

**Authors:** Yashvee Dunneram, Darren Charles Greenwood, Victoria J Burley, Janet E Cade

**Affiliations:** 1 Nutritional Epidemiology Group, School of Food Science and Nutrition, University of Leeds, Leeds, UK; 2 Division of Epidemiology and Biostatistics, University of Leeds, Leeds, UK

**Keywords:** natural menopause, diet, food groups, nutrients, UKWCS

## Abstract

**Background:**

Age at natural menopause is a matter of concern for women of reproductive age as both an early or late menopause may have implications for health outcomes.

**Methods:**

Study participants were women aged 40–65 years who had experienced a natural menopause from the UK Women’s Cohort Study between baseline and first follow-up. Natural menopause was defined as the permanent cessation of menstrual periods for at least 12 consecutive months. A food frequency questionnaire was used to estimate diet at baseline. Reproductive history of participants was also recorded. Regression modelling, adjusting for confounders, was used to assess associations between diet and age at natural menopause.

**Results:**

During the 4-year follow-up period, 914 women experienced a natural menopause. A high intake of oily fish and fresh legumes were associated with delayed onset of natural menopause by 3.3 years per portion/day (99% CI 0.8 to 5.8) and 0.9 years per portion/day (99% CI 0.0 to 1.8), respectively. Refined pasta and rice was associated with earlier menopause (per portion/day: −1.5 years, 99% CI −2.8 to −0.2). A higher intake of vitamin B6 (per mg/day: 0.6 years, 99% CI 0.1 to 1.2) and zinc (per mg/day: 0.3 years, 99% CI −0.0 to 0.6) was also associated with later age at menopause. Stratification by age at baseline led to attenuated results.

**Conclusion:**

Our results suggest that some food groups (oily fish, fresh legumes, refined pasta and rice) and specific nutrients are individually predictive of age at natural menopause.

## Introduction

The average age of menopause in the UK is reported to be 51 years.^1^ Menopause is an important phase in a woman’s life indicating the end of the reproductive life span with reduction in oestrogen and increased progesterone levels.^2 3^ Several studies have documented an association between earlier age at natural menopause and lower bone density, osteoporosis, depression and premature death.^4 5^ Other studies have shown increased risk of cardiovascular and coronary diseases.^6 7^ In contrast, a late menopause has been associated with a higher risk for breast, ovarian and endometrial cancers.^8^

A number of causes have been postulated for the relationship between age at menopause and these health outcomes, such as genetic factors, behavioural and environmental exposures, socio-demographic factors, hormonal mechanisms and health-related factors.^9^ Diet can also be an underlying factor.^9^ Two large cohort studies have also hypothesised an association^10 11^ but reported conflicting findings.

The limited number of studies and contradictory results^10–12^ in this area suggests the need for further cohort studies with detailed dietary intake measures to clarify this association. The aim of this analysis was to explore the associations between food groups and nutrient intake in a large cohort of British women with age at incident natural menopause. We hypothesised that intake of healthier food groups such as fruits and vegetables would be associated with an earlier menopause while a high consumption of meat and processed meat would delay the onset of menopause.

## Methods

### Study population

The UK Women’s Cohort Study (UKWCS) is a large prospective study consisting of 35 372 women aged between 35 and 69 years. Recruited participants were from England, Scotland and Wales.^13^ Baseline data were collected between the years 1995 and 1998 via postal questionnaire. Follow-up data were collected on average 4 years later, between the years 1999 and 2002.^13^

### Study design and data collection

**Figure 1:**
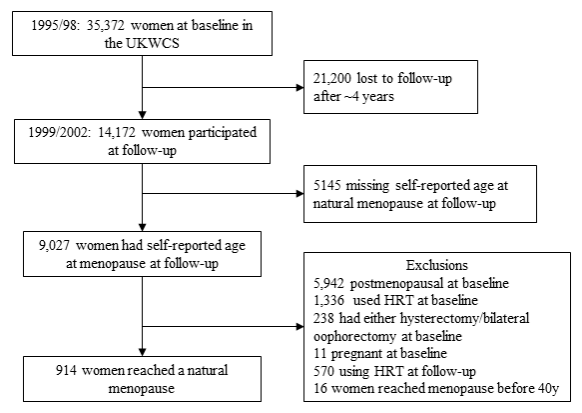
Flow chart of participants included in analysis diet and age at natural menopause. HRT, hormone replacement therapy; UKWCS, UK Women’s Cohort Study.

In total, 14 172 women who participated at both baseline and follow-up were considered for this study. Information was collected on demographic details, weight history, physical activity, reproductive history (age at last period; number of periods in last 12 months; use of hormone replacement therapy (HRT)), anthropometric and other health-related factors at baseline as well as at follow-up. Participants who experienced a natural menopause at follow-up were identified through comparison of baseline and follow-up data. Natural menopause was defined as the permanent cessation of the menstrual periods for at least 12 consecutive months.^2^ Menstruating women, that is, those having one or more menstrual period in the last 12 months at baseline and who became naturally postmenopausal at follow-up were included in the final analysis. Inclusion criteria also comprised never used HRT at baseline and currently not using HRT at follow-up (as HRT use may influence the bleeding pattern among premenopausal women^14^). Women who ever used HRT after reaching menopause at phase II were also included. Women who had bilateral oophorectomy and hysterectomy at baseline as well as pregnant women at baseline were excluded from the study. In addition, only women with an age at natural menopause between ≥40 and ≤65 years were included (as no menstruation before the age of 40 might be chemically induced or due to surgical procedures). In addition, participants with missing data on the main study outcome, age at natural menopause and confounders were also excluded from the study (figure 1).

### Dietary assessment

Dietary assessment at baseline involved a detailed 217-item food frequency questionnaire (FFQ) derived from the FFQ which was validated on a subsample of 303 cohort subjects against a 4-day food diary as well as fasting blood measures of specific nutrients.^13 15 16^ Using the different frequency categories of the FFQ, the number of daily portions for the 217 food items was defined. These were consequently converted into weight of each food consumed per day based on the Food Standards Agency portion sizes book.^17^ For the current study, the individual food items were collated into food groups according to their culinary uses (eg, Mediterranean vegetables, cruciferous vegetables, citrus family fruits) and nutrient profile (eg, fat or fibre content) (online supplementary table 1). In total, 15 food items were considered individually. Seven food items were considered individually due to their specific nutrient profile such as textured vegetable proteins, oily fish, shellfish, grapes, herbal tea, tea and wines, which have antioxidant properties and might separately affect age at natural menopause. The remaining eight food items (eg, tomatoes, sauces, low calorie salad cream, etc) were considered indivdually because they could not be collated under any of the other food groups. Furthermore, in order to have a better estimate for the difference in mean age at natural menopause across the different food groups, results were presented per portion size.

10.1136/jech-2017-209887.supp1 Supplementary file 1

### Covariate assessment

A directed acyclic graph (DAG) (online supplementary figure 1) with diet (food groups) as the main exposure and age at natural menopause (continuous) as the outcome was generated to determine confounding variables. Based on available literature and data collected, potential confounding variables (age, parity, energy intake, body mass index (BMI), social class, age at first full-term pregnancy, age at menarche, smoking, alcohol consumption and physical activity) were included in the DAG. According to the minimal sufficiency set of adjustments, physical activity (MET-hours/week), smoking status (current vs not current smoker), alcohol consumption (g/day) and social class (routine and manual, intermediate, professional and managerial) were identified as confounders and were thus adjusted for in the regression models. For the associations between nutrient intake and age at natural menopause, total energy intake was also adjusted for the non-energy-containing nutrients (women were excluded if they had extremely high (>6000 kcal/day) or low (<500 kcal/day) energy intake) and energy from other macronutrients was adjusted for specific macronutrients.

### Statistical analysis

Descriptive statistics were used to explore the socio-demographic and obstetric history of the women. Linear regression models were used to determine the relationships between the various food groups (continuous exposure in g/day) as well as nutrients (continuous exposure) and age at natural menopause (continuous outcome in years). In addition, because younger women at baseline have less chance of a later menopause we evaluated the associations by stratifying on age at baseline (≤50 vs >50 years). An estimate >0 was considered as a later age at natural menopause. Assumptions for linear regression were checked by plotting the residuals against fitted values which showed a constant variance and a histogram demonstrated a normal distribution of the residuals. Due to the differences in age at natural menopause by vegetarian status and parity as evidenced by previous studies,^10 18^ sensitivity analysis exploring that relationship was undertaken stratified by vegetarian status and parity (nulliparous vs multiparous). Moreover, since presence of diabetes might influence both diet and age at natural menopause, we also adjusted for diabetes. To take account of multiple testing, the significance level was set at 1% with 99% CIs. All analyses were conducted using Stata V. 14.0 (StataCorp).

## Results

### Socio-demographic characteristics

Of the 1874 women who were premenopausal at baseline survey (and had self-reported age at natural menopause at follow-up), 914 had become postmenopausal at 4-year follow-up. Baseline characteristics of the participants are outlined in table 1. The mean age at natural menopause at follow-up was 50.5 years (95% CI 50.3 to 50.8). Participants had a mean BMI of 23.9 kg/m^2^ (95% CI 23.6 to 24.1) and 9.6% were categorised as obese. Physical activity level was quite low among the participants with a mean of 15 min/day. This study also included 38% vegetarian participants. Most of the women were married, parous and of professional and managerial class. In this study, only 8% of women smoked and the mean alcohol consumption was 9 g/day (around one unit).

**Table 1:** Baseline characteristics of participants

Characteristics (mean/%, 95% CI)	Age at natural menopause			
	40–48 years n=226	49–51 years n=319	≥52 years n=369	Total n=914
Age at baseline (years)	45.4 (45.0 to 45.8)	49.0 (48.8 to 49.2)	52.1 (51.9 to 52.4)	49.4 (49.2 to 49.6)
Birth year (years)	1950 (1950 to 1951)	1947 (1946 to 1947)	1944 (1943 to 1944)	1946 (1946 to 1946)
Body mass index (kg/m²)	23.6 (23.0 to 24.1)	23.5 (23.1 to 23.9)	24.3 (23.8 to 24.7)	23.8 (23.6 to 24.1)
Obese>30 kg/m² (%)	8.4 (5.4 to 12.8)	6.9 (4.6 to 10.3)	12.6 (9.5 to 16.4)	9.6 (7.8 to 11.7)
Physical activity (min/day)	12.8 (10.2 to 15.4)	16.5 (13.6 to 19.4)	14.1 (11.8 to 16.3)	14.6 (13.1 to 16.1)
Vegetarian (%)	45.7 (39.2 to 52.4)	44.2 (38.8 to 49.8)	33.0 (28.3 to 38.0)	40.0 (36.9 to 43.3)
Alcohol consumption (g/day)	10.1 (8.4 to 11.8)	8.6 (7.5 to 9.7)	8.4 (7.4 to 9.4)	9.0 (8.2 to 9.6)
Smoking (%)	10.2 (6.9 to 14.9)	8.0 (5.4 to 11.5)	5.0 (3.1 to 7.7)	7.3 (5.8 to 9.2)
Age at menarche (years)	12.6 (12.4 to 12.8)	12.7 (12.5 to 12.8)	12.9 (12.7 to 13.1)	12.7 (12.6 to 12.8)
Age at first full-term pregnancy (years)	26.6 (25.8 to 27.5)	26.0 (25.4 to 26.5)	25.6 (25.1 to 26.0)	25.9 (25.6 to 26.3
Parous (%)	68.6 (62.0 to 74.5)	77.1 (72.1 to 81.5)	84.3 (80.1 to 87.8)	78.0 (75.1 to 80.4)
Ever married (%)	76.3 (70.3 to 81.5)	78.6 (73.7 to 82.8)	78.1 (73.5 to 82.0)	77.8 (75.0 to 81.1)
Degree level (%)	36.7 (30.5 to 43.4)	37.5 (32.2 to 43.1)	26.1 (21.7 to 31.0)	32.8 (29.7 to 36.0)
Professional and managerial class (%)	70.0 (63.6 to 75.6)	63.8 (58.3 to 68.9)	60.3 (55.1 to 65.2)	63.9 (60.7 to 67.0)

### Association between food groups and age at natural menopause

An increase in portion size of refined pasta and rice as well as savoury snacks was associated with an earlier age at natural menopause by 1.8 years (99% CI −3.0 to −0.5) and 0.9 years (99% CI −1.7 to −0.1), respectively in the unadjusted model (table 2). In the adjusted model, for each additional portion of oily fish and fresh legumes, age at menopause was increased by 3.3 years (99% CI 0.8 to 5.8) and 0.9 years (99% CI 0.0 to 1.8), respectively. On the other hand, a higher intake of refined pasta and rice (per portion/day: 1.5 years; 99% CI −2.8 to −0.2) was associated with an earlier menopause. Stratification by age at baseline led to reduced associations between the various food groups and age at natural menopause. The CIs were wider because of the smaller samples in these subgroups.

**Table 2:** Estimates (overall and stratified on age at baseline) for the association between daily intake of the food groups/portion size (g) and age at natural menopause (years)

Daily intake/portion size	Age at baseline						≤50 years			>50 years		
	Estimate*	99% CI	P values	Estimate†	99% CI	P values	Estimate‡	99% CI	P values	Estimate§	99% CI	P values
Starchy food sources												
Wholegrain products/33 g	0.0	−0.1 to 0.2	0.491	0.0	−0.1 to 0.2	0.443	0.0	−0.2 to 0.1	0.559	0.1	−0.0 to 0.3	0.034
Refined grain products/51 g	−0.0	−0.5 to 0.3	0.488	−0.2	−0.5 to 0.2	0.267	−0.1	−0.6 to 0.3	0.495	−0.3	−0.7 to 0.0	0.017
Low-fibre breakfast cereals/40 g	0.0	−1.0 to 1.0	0.920	−0.1	−1.1 to 1.0	0.888	−0.7	−1.8 to 0.4	0.109	0.5	−0.5 to 1.5	0.163
High-fibre breakfast cereals/85 g	0.2	−0.2 to 0.6	0.136	0.2	−0.3 to 0.7	0.273	0.1	−0.4 to 0.6	0.621	0.0	−0.4 to 0.5	0.915
Plain potatoes/210 g	0.4	−0.4 to 1.1	0.213	0.5	−0.3 to 1.2	0.114	−0.1	−1.0 to 0.9	0.868	−0.2	−0.8 to 0.5	0.516
Potatoes with added fat/127 g	0.3	−1.1 to 1.8	0.566	0.1	−1.4 to 0.2	0.829	−0.1	−1.8 to 1.7	0.929	0.1	−1.4 to 1.6	0.843
Refined pasta and rice/210 g	−1.8	−3.0 to −0.5	<0.001	−1.5	−2.8 to −0.2	0.003	−0.9	−2.3 to 0.5	0.101	0.8	−0.7 to 2.2	0.166
Wholegrain pasta and rice/197 g	0.4	−1.0 to 1.7	0.492	0.5	−0.9 to 2.0	0.309	0.0	−1.7 to 1.6	0.958	0.6	−0.7 to 1.9	0.243
Protein and fat food sources												
Low-fat dairy products/80 g	0.0	−0.1 to 0.1	0.043	0.0	−0.1 to 0.1	0.700	−0.1	−0.2 to 0.0	0.053	0.0	−0.1 to 0.1	0.835
High-fat dairy products/75 g	−0.1	−0.2 to 0.1	0.279	−0.2	−0.2 to 0.1	0.323	−0.1	−0.3 to 0.1	0.493	−0.1	−0.3 to 0.1	0.118
Butter and hard margarine/10 g	0.1	−0.2 to 0.4	0.350	0.2	−0.2 to 0.5	0.228	0.1	−0.3 to 0.5	0.475	0.0	−0.3 to 0.3	0.838
Margarine/9 g	−0.2	−0.4 to 0.1	0.103	−0.2	−0.5 to 0.1	0.101	−0.1	−0.4 to 0.2	0.636	0.0	−0.3 to 0.3	0.958
Low-fat spreads/7 g	0.1	−0.2 to 0.4	0.264	0.1	−0.2 to 0.4	0.538	0.1	−0.3 to 0.5	0.628	−0.1	−0.4 to 0.2	0.357
High-fat dressing/23 g	−0.1	−1.2 to 0.9	0.717	−0.0	−1.0 to 1.0	0.993	0.2	−1.0 to 1.3	0.708	0.0	1.0 to 1.1	0.932
Low-fat dressing/30 g	1.3	−0.8 to 3.4	0.116	0.8	−1.3 to 2.9	0.309	0.8	−1.6 to 3.1	0.401	−0.4	−2.5 to 1.7	0.596
Soya bean products/62 g	−0.0	−0.1 to 0.1	0.978	−0.0	−0.2 to 0.1	0.812	0.0	−0.1 to 0.2	0.392	−0.1	−0.3 to 0.1	0.136
Textured vegetable protein/130 g	−4.2	−13.1 to 4.7	0.226	−3.6	−12.6 to 5.4	0.300	−2.9	−12.1 to 6.3	0.414	−2.7	−13.0 to 7.7	0.506
Pulses/91 g	−0.4	−1.1 to 0.2	0.087	−0.3	−1.0 to 0.4	0.230	0.1	−0.7 to 0.8	0.760	−0.5	−1.1 to 0.2	0.065
Eggs/eggs dishes/88 g	1.0	−0.4 to 2.4	0.070	0.6	−0.9 to 2.0	0.301	−0.4	−2.0 to 1.2	0.536	−0.5	1.9 to 0.9	0.358
Fish and fish dishes/140 g	1.4	−0.6 to 3.4	0.068	1.2	−0.9 to 3.3	0.130	−1.0	−3.2 to 1.3	0.264	1.4	−0.7 to 3.6	0.085
Oily fish/90 g	3.2	0.8 to 5.6	0.001	3.3	0.8 to 5.8	0.001	1.9	−1.2 to 4.9	0.118	0.9	−1.3 to 3.1	0.311
Shell fish/60 g	1.7	−4.4 to 7.8	0.462	2.2	−4.1 to 8.5	0.361	−4.0	−11.5 to 3.5	0.165	1.7	4.1 to 7.6	0.438
Red meat/189 g	1.9	0.3 to 3.5	0.003	1.5	−0.2 to 3.2	0.021	−0.2	−2.2 to 1.8	0.830	0.9	−0.6 to 2.5	0.123
Processed meat/74 g	1.3	−0.4 to 3.0	0.042	1.0	−0.8 to 2.7	0.150	0.2	−1.8 to 2.2	0.830	0.4	−1.2 to 2.1	0.495
Poultry/143 g	1.6	−0.6 to 3.8	0.063	1.4	−0.9 to 3.6	0.109	0.0	−2.4 to 2.4	0.993	1.2	−1.2 to 3.6	0.186
Offal/100 g	6.9	−2.2 to 16.1	0.051	5.9	−3.5 to 15.2	0.104	−2.0	−14.4 to 10.4	0.675	−0.2	−8.1 to 7.7	0.948
Vegetables												
Vegetable dishes/214 g	−0.6	−1.3 to 0.2	0.069	−0.5	−1.3 to 0.3	0.102	−0.7	−1.7 to 0.2	0.055	−0.3	−1.0 to 0.5	0.341
Allium/39 g	0.3	−0.5 to 1.2	0.322	0.5	−0.4 to 1.4	0.125	0.1	−1.0 to 1.1	0.814	−0.2	−1.1 to 0.6	0.478
Fresh legumes/75 g	1.0	0.1 to 1.8	0.003	0.9	0.0 to 1.8	0.007	0.0	−0.9 to 1.0	0.896	0.4	−0.4 to 1.2	0.205
Mediterranean vegetables/60 g	−0.0	−0.6 to 0.6	1.000	0.1	−0.5 to 0.6	0.730	0.1	−0.5 to 0.7	0.597	0.2	−0.4 to 0.8	0.363
Salad vegetables/43 g	0.4	−0.0 to 0.8	0.021	0.4	−0.0 to 0.9	0.018	0.4	−0.1 to 0.8	0.036	0.2	−0.4 to 0.7	0.441
Cruciferous vegetables/75 g	0.3	−0.0 to 0.6	0.017	0.3	−0.0 to 0.7	0.024	0.0	−0.3 to 0.4	0.845	0.0	−0.4 to 0.4	0.969
Tomatoes/83 g	0.2	−0.4 to 0.8	0.352	0.0	−0.6 to 0.7	0.855	0.1	−0.6 to 0.8	0.765	0.0	−0.6 to 0.5	0.822
Mushrooms/34 g	0.3	−0.8 to 1.5	0.431	0.3	−0.9 to 1.4	0.581	−0.3	−1.7 to 1.0	0.543	0.1	−1.1 to 1.2	0.860
Roots and tubers/66 g	0.4	−0.1 to 1.0	0.032	0.4	−0.1 to 0.9	0.057	0.1	−0.5 to 0.7	0.715	0.4	−0.2 to 1.0	0.102
Fruits												
Stone fruits/49 g	0.5	−0.2 to 1.3	0.058	0.4	−0.3 to 1.2	0.155	0.0	−0.7 to 0.8	0.884	0.3	−0.6 to 1.1	0.442
Deep orange and yellow fruits/118 g	0.6	−0.1 to 1.3	0.036	0.5	−0.2 to 1.3	0.051	0.1	−0.6 to 0.9	0.669	0.5	−0.2 to 1.3	0.079
Grapes/100 g	0.8	−0.1 to 1.6	0.022	0.7	−0.2 to 1.6	0.039	−0.3	−1.5 to 0.9	0.546	0.2	−0.5 to 0.9	0.428
Citrus family fruits/92 g	0.3	−0.2 to 0.9	0.149	0.2	−0.3 to 0.8	0.316	−0.2	−0.8 to 0.5	0.542	−0.1	−0.6 to 0.5	0.799
Rhubarb/130 g	0.8	−0.6 to 2.2	0.143	0.7	−0.7 to 2.1	0.181	0.7	−0.8 to 2.2	0.233	0.0	−1.4 to 1.3	0.937
Berries/48 g	0.3	−0.2 to 0.8	0.151	0.2	−0.3 to 0.7	0.233	−0.1	−0.7 to 0.5	0.733	0.0	−0.5 to 0.4	0.839
Bananas/100 g	0.1	−0.4 to 0.6	0.718	0.0	−0.5 to 0.6	0.893	−0.1	−0.8 to 0.6	0.668	−0.4	−0.9 to 0.2	0.073
Pomes/116 g	0.1	−0.3 to 0.4	0.670	0.0	−0.3 to 0.4	0.805	0.0	−0.4 to 0.4	0.867	−0.1	−0.4 to 0.3	0.586
Dried fruits/28 g	0.4	−0.0 to 0.9	0.016	0.4	−0.0 to 0.9	0.017	0.4	−0.2 to 0.9	0.072	−0.1	−0.5 to 0.6	0.734
Other food groups												
Sauces/83 g	0.4	−2.0 to 2.7	0.691	0.1	−2.3 to 2.5	0.910	−1.0	−4.0 to 1.9	0.357	−0.7	−2.9 to 1.6	0.441
Pickles/chutneys/35 g	−0.1	−1.4 to 1.2	0.822	−0.2	−1.5 to 1.1	0.743	0.0	−1.5 to 1.4	0.957	0.3	−1.1 to 1.6	0.601
Soups/163 g	0.9	−0.2 to 2.0	0.035	0.9	−0.2 to 2.0	0.038	0.3	−1.1 to 1.7	0.587	0.4	−0.6 to 1.4	0.301
Confectionery and spreads/44 g	0.0	−0.3 to 0.3	0.950	−0.0	−0.3 to 0.3	0.867	−0.1	−0.5 to 0.3	0.484	0.0	−0.3 to 0.3	0.891
Nuts and seeds/24 g	0.1	−0.3 to 0.5	0.449	0.1	−0.2 to 0.5	0.421	0.1	−0.2 to 0.5	0.368	−0.1	−0.5 to 0.3	0.376
Savoury snacks/26 g	−0.9	−1.7 to −0.1	0.006	−0.9	−1.8 to 0.1	0.017	−0.5	−1.5 to 0.5	0.196	−0.7	−1.6 to 0.3	0.075
Biscuits/15 g	−0.1	−0.5 to 0.2	0.297	−0.2	−0.5 to 0.2	0.155	−0.2	−0.6 to 0.2	0.232	−0.2	−0.5 to 0.2	0.211
Cakes/66 g	0.3	−1.1 to 1.6	0.592	−0.0	−1.6 to 1.5	0.934	−0.8	2.5 to 0.9	0.220	0.7	−0.8 to 2.3	0.226
Pastries and puddings/84 g	−0.3	−1.4 to 0.7	0.402	−0.3	−1.5 to 0.8	0.413	−0.8	−2.1 to 0.5	0.121	−0.5	−1.6 to 0.5	0.182
Drinks and beverages												
Tea/260 g	−0.1	−0.2 to 0.1	0.148	−0.1	−0.3 to 0.0	0.042	−0.1	−0.3 to 0.1	0.103	0.0	0.2 to 0.1	0.450
Herbal tea/260 g	0.1	−0.3 to 0.4	0.648	0.1	−0.2 to 0.4	0.415	0.1	−0.2 to 0.5	0.298	0.0	−0.3 to 0.3	0.967
Coffee/190 g	0.0	−0.1 to 0.2	0.470	0.1	−0.1 to 0.2	0.249	0.0	−0.2 to 0.2	0.842	0.0	−0.2 to 0.1	0.641
Other hot beverages/23 g	0.1	−0.4 to 0.5	0.742	0.1	−0.4 to 0.6	0.650	0.0	−0.6 to 0.6	0.995	−0.2	−0.7 to 0.3	0.299
Juices/145 g	0.2	−0.2 to 0.6	0.243	0.1	−0.3 to 0.6	0.400	0.0	−0.5 to 0.5	0.896	0.1	−0.3 to 0.5	0.448
Soft drinks/111 g	−0.7	−1.5 to 0.1	0.022	−0.8	−1.6 to 0.1	0.016	−0.5	−1.3 to 0.3	0.085	0.0	−1.1 to 1.1	0.988
Low calorie/diet soft drinks/161 g	−0.1	−0.7 to 0.4	0.516	−0.1	−0.7 to 0.5	0.566	−0.2	−1.0 to 0.5	0.431	−0.2	−0.7 to 0.3	0.333
Wines/1 g	−0.2	−0.6 to 0.3	0.275	0.1	−0.5 to 0.8	0.563	0.1	−0.6 to 0.7	0.768	−0.3	−1.1 to 0.5	0.325
Beer and cider/1 g	−0.5	−1.1 to 0.2	0.053	−0.5	−1.3 to 0.3	0.093	0.0	−0.7 to 0.7	0.871	−0.2	−1.7 to 1.3	0.690
Port, sherry, liqueurs/1 g	0.9	−0.6 to 2.5	0.112	1.1	−0.5 to 2.7	0.068	1.1	−0.8 to 3.1	0.139	0.4	−1.0 to 1.8	0.420
Spirits/1 g	−0.3	−1.1 to 0.4	0.215	−0.1	−1.0 to 0.7	0.686	−0.1	−0.9 to 0.7	0.668	0.4	−0.7 to 1.5	0.368

*Difference in age at natural menopause, unadjusted model (n=914).

†Difference in age at natural menopause, model adjusted for the following factors: physical activity level, alcohol consumption, smoking, social class (n=838).

‡Difference in age at natural menopause for those aged 50 years or below in the fully adjusted model (n=477).

§Difference in age at natural menopause for those aged above 50 years in the fully adjusted model (n=361).

For the association between nutrients and age at natural menopause, a later age at natural menopause by approximately 0.6 years was found with a higher intake of vitamin B6 per mg (99% CI 0.1 to 1.2). Similarly, a higher intake of zinc was associated with a delayed age at natural menopause by 0.3 years per mg (99% CI −0.0 to 0.6) (table 3). Stratification by age at baseline further demonstrated that a higher intake of carbohydrates was associated with an earlier age at natural menopause by 0.2 years (99% CI −0.4 to −0.0) among women 50 years or below.

**Table 3:** Estimates (overall and stratified on age at baseline) for the association between daily nutrient intake and age at natural menopause (years)

Daily nutrient intake	Age at baseline						≤50 years			>50 years		
	Estimate*	99% CI	P values	Estimate†	99% CI	P values	Estimate‡	99% CI	P values	Estimate§	99% CI	P values
Fibre (g)	0.0	−0.0 to 0.1	0.111	−0.0	−0.1 to 0.0	0.087	0.0	−0.0 to 0.1	0.161	0.0	−0.0 to 0.0	0.641
% energy from fats	0.0	−0.1 to 0.0	0.140	−0.1	−0.4 to 0.1	0.144	−0.2	−0.4 to 0.0	0.010	−0.1	−0.4 to 0.2	0.356
% energy from proteins	0.1	0.0 to 0.2	0.005	−0.0	−0.3 to 0.2	0.713	−0.3	−0.5 to 0.0	0.011	−0.0	−0.3 to 0.3	0.995
% energy from carbohydrates	0.0	−0.0 to 0.1	0.416	−0.1	−0.3 to 0.1	0.227	−0.2	−0.4 to −0.0	0.009	−0.1	−0.3 to 0.2	0.508
% energy from saturated fats	−0.1	−0.2 to 0.0	0.094	−0.1	−0.2 to 0.1	0.171	−0.1	−0.3 to 0.1	0.155	−0.0	−0.2 to 0.1	0.443
% energy from polyunsaturated fats	−0.1	−0.2 to 0.1	0.243	−0.0	−0.2 to 0.2	0.941	0.1	−0.2 to 0.3	0.485	0.0	−0.2 to 0.2	0.936
% energy from monounsaturated fats	0.0	−0.2 to 0.1	0.324	−0.1	−0.2 to 0.4	0.488	0.0	−0.3 to 0.4	0.795	0.0	−0.3 to 0.3	0.855
Vitamin C (mg)	0.0	0.0 to 0.1	0.010	0.0	−0.0 to 0.1	0.031	0.0	−0.0 to 0.1	0.329	0.0	−0.0 to 0.1	0.585
Vitamin B1 (mg)	0.0	−0.2 to 0.1	0.271	−0.1	−0.2 to 0.0	0.110	−0.0	−0.2 to 0.1	0.396	−0.1	−0.2 to 0.0	0.130
Vitamin B2 (mg)	0.3	−0.1 to 0.6	0.060	0.3	−0.2 to 0.9	0.105	−0.2	−0.9 to 0.4	0.306	0.0	−0.5 to 0.5	0.987
Vitamin B6 (mg)	0.4	−0.0 to 0.7	0.014	0.6	0.1 to 1.2	0.005	0.0	−0.6 to 0.7	0.900	0.2	−0.4 to 0.8	0.508
Vitamin B12 (µg)	0.0	−0.0 to 0.0	0.198	0.0	−0.0 to 0.0	0.440	0.0	−0.0 to 0.0	0.848	0.0	−0.0 to 0.0	0.536
Folate (µg)	0.1	−0.0 to 0.2	0.038	0.2	−0.0 to 0.3	0.029	0.0	−0.2 to 0.2	0.805	0.1	−0.1 to 0.2	0.408
Vitamin D (µg)	0.4	−0.0 to 0.7	0.011	0.4	−0.0 to 0.8	0.017	0.2	−0.3 to 0.7	0.281	0.1	−0.3 to 0.5	0.519
Vitamin A (µg)	0.1	0.0 to 0.2	0.008	0.1	−0.0 to 0.2	0.020	0.0	−0.1 to 0.1	0.795	0.0	−0.1 to 0.1	0.675
Vitamin E (mg)	0.0	−0.1 to 0.1	0.516	−0.1	−0.1 to 0.0	0.145	0.0	−0.1 to 0.1	0.377	−0.0	−0.1 to 0.1	0.391
Calcium (mg)	0.0	−0.1 to 0.2	0.564	−0.0	−0.2 to 0.2	0.791	−0.2	−0.5 to 0.1	0.042	−0.1	−0.3 to 0.1	0.423
Iron (mg)	0.1	−0.0 to 0.2	0.085	0.1	−0.0 to 0.2	0.044	0.1	−0.1 to 0.2	0.244	0.0	−0.1 to 0.1	0.705
Zinc (mg)	0.2	−0.0 to 0.3	0.012	0.3	−0.0 to 0.6	0.007	−0.0	−0.4 to 0.3	0.725	0.2	−0.1 to 0.5	0.081

*Difference in age at natural menopause, unadjusted model (n=910).

†Difference in age at natural menopause, model adjusted for the following factors: physical activity level, alcohol consumption, smoking, social class, total energy intake (n=838).

‡Difference in age at natural menopause for those aged 50 years or below in the fully adjusted model (n=477).

§Difference in age at natural menopause for those aged above 50 years in the fully adjusted model (n=361).

### Sensitivity analysis

Our findings demonstrated that non-vegetarians reach a natural menopause 0.8 years later compared with vegetarians (99% CI 0.2 to 1.4). Exploring associations for non-vegetarians alone showed they had an earlier age at natural menopause associated with an increased consumption of savoury snacks (per portion/day: −1.7 years, 99% CI −3.1 to −0.4) and soft drinks (per portion/day: −1.3 years, 99% CI −2.5 to −0.2) while an increase in intake of oily fish (per portion/day: 3.4 years, 99% CI 0.2 to 6.5) and fresh legumes (per portion/day: 1.4 years, 99% CI 0.2 to 2.7) were associated with a later onset of menopause (online supplementary table 2).

Sensitivity analysis by parity demonstrated a difference for the association between the various food groups and age at natural menopause for nulliparous against the multiparous participants. In multiparous women, a later onset of age at natural menopause was found to be associated with an increased intake of oily fish (per portion/day: 3.3 years, 99% CI 0.3 to 6.3) and fresh legumes (per portion/day: 1.1 years, 99% CI 0.1 to 2.01) while an increase in intake of refined pasta and rice (per portion/day: −1.9 years, 99% CI −3.3 to −0.4) as well as savoury snacks (per portion/day: −1.0 years, 99% CI −2.1 to −0.0) was associated with an earlier age at natural menopause. For nulliparous women, a higher consumption of grapes (per portion/day: 2.5 years, 99% CI 0.0 to 4.9) and poultry (per portion/day: 5.2 years, 99 % CI 0.1 to 10.3) was found to be significantly associated with a later age at natural menopause (online supplementary table 3).

Further adjusting the model by presence of diabetes demonstrated no changes in our results (online supplementary table 4).

## Discussion

This is the first study of women in the UK to report on food and nutrient intake in relation to age at incidence of natural menopause. Of 14 172 women who were followed up for approximately 4 years, 914 women went through a natural menopause. The mean age at natural menopause was 50.5 years with a median age of 51 years. We found that intakes of oily fish and fresh legumes were associated with later age at menopause and intake of refined pasta/rice was associated with an earlier menopause. Only a few previous studies have reported diet in relation to age at natural menopause with a limited number of food items/groups included.^10 12^ Previous research has mainly been focused on the relationship between socio-demographic as well as lifestyle factors (education status, marital status, parity, etc) and age at natural menopause.^18–21^

Our results demonstrate that each additional increment in fresh legumes portion/day was associated with a later age at natural menopause by 0.9 years. Fresh legumes are a good source of antioxidants, which can partly explain this association. This has been supported by the biochemical and molecular analyses undertaken by Matamoros *et al*.^22^ Similarly, in a Japanese prospective study the antioxidant properties of green and yellow vegetables were postulated for the association between a higher intake of the green and yellow vegetables and a later age at natural menopause.^12^ Oocyte maturation, ovulation, luteolysis and follicle atresia are affected by reactive oxygen species (ROS). Phenolic compounds, vitamins and carotenoids in vegetables counteract the ROS and may thus decrease the proportion of follicles undergoing follicular atresia.^23 24^ Further support of this theory from our findings was a later age at natural menopause with a high intake of vitamin B6 and zinc as both of these have antioxidant properties.^23 25^ Likewise, Stepaniack *et al* 26 demonstrated an association between use of vitamin and mineral supplements and a later menopause.

Our findings demonstrate a later age at natural menopause by approximately 3 years for each additional portion/day of oily fish. However, in contrast to our findings, a recent review article as well as a 10-year follow-up study reported an earlier onset of menopause with high intake of polyunsaturated fats.^27 28^ Nagel *et al* 10 reported no association between fish intake and age at natural menopause but it was not clear if oily fish was considered separately. Oily fish is a rich source of the omega-3 fatty acid which can potentially improve antioxidant capacity.^29^ Therefore, in a similar way to the fresh legumes and vitamins described above, the antioxidant properties exerted by the oily fish intake could possibly offset ROS, therefore decreasing the proportion of follicles undergoing follicular atresia and delaying onset of natural menopause.

In the present study, increasing refined pasta and rice consumption was associated with an earlier age at natural menopause. The EPIC-Heidelberg study also reported a similar association.^10^ High consumption of refined carbohydrates (classified as high glycaemic index foods) increases the risk of insulin resistance. Insulin resistance can lead to decreased sex hormone binding globulin levels (SHBG) as a result of the inhibitory effect of insulin on the SHBG production in the liver^30^ as well as increased oestrogen levels.^31^ High oestrogen levels cause release of the luteinising hormones which triggers ovulation, which might imply more cycles and rapid depletion of oocytes, consequently leading to an earlier menopause.^32^ This can be supported by a recent review study which reported that women with type II diabetes mellitus tend to have an earlier menopause although additional evidence is required to clarify this association.^33^

Although we found that fresh legumes are associated with a later menopause, our study further demonstrated that women who were vegetarian had an earlier age at natural menopause compared with non-vegetarians. This finding is in line with other studies which also reported an earlier age at natural menopause among vegetarians.^34 35^ The vegetarian diet, which normally consist of high fibre and no animal fat-containing foods, may affect the levels of the luteinising hormone, follicle stimulating hormone and the length of the menstrual cycle.^36^ Previous studies have demonstrated that high fibre and decreased fat intakes were both associated with a lower oestrogen level, which may account for the earlier age at natural menopause among vegetarians.^37 38^ However, caution should be taken in interpreting this finding as vegetarian status was self-reported in this study.

It is possible that results for younger women may differ from those for older women. This could result from different diets between younger and older women,^39^ and that younger women have less opportunity to report a later menopause. To explore this, stratifying on age at baseline showed reduced associations within each subgroup.

This is the first study looking prospectively at the relationship between diet and age at natural menopause in the UK. Strengths of this study include the investigation of the association between individual nutrients and a wide variety of food groups and age at natural menopause compared with similar few previous studies. Careful adjustment for likely confounders was also carried out in the regression modelling using the DAG. A limitation of this prospective cohort study is that diet was reported by the participants using an FFQ and may thus be subjected to recall bias. However, FFQ enables recording of a long-term diet, thus showing its cumulative influence on the outcome while food diaries/24-hour recall give only a snapshot of the diet. Our sample was also more health conscious given the high number of vegetarians in our sample population and more well-off participants than the general population as shown in the descriptive table (table 1). However, our study still includes women from a range of different background which implies that findings of this study may be extrapolated to other countries.

Women with an earlier menopause spend more years deprived from the benefits of oestrogen compared with women who become menopausal around the normal menopausal age range, which puts them at a greater risk of some future poor health outcomes such as osteoporosis and heart disease. On the other hand, women with a later onset of menopause are at greater risk of breast, endometrial and ovarian cancers. Our findings confirm that diet may be associated with the age at natural menopause. This may be relevant at a public health level since age at natural menopause may have implications on future health outcomes. Health practitioners might thus also need to take into account the diet of women when dealing with menopause-related issues.

In summary, our study is the first to demonstrate that diet is associated with age at natural menopause in a large cohort of British women. Intakes of oily fish and fresh legumes were found to be associated with a later onset of natural menopause while higher intake of refined pasta and rice was associated with younger age at natural menopause. The nutrients vitamin B6 and zinc were also found to be associated with a later age at natural menopause. Women who were vegetarian had an earlier age at natural menopause compared to non-vegetarians.

**What is already known on this subject**

- Several factors including socio-demographic and reproductive factors are associated with age at natural menopause. Limited existing studies present conflicting evidence between diet and age at natural menopause.

**What this study adds**

- This is the first study to our knowledge which demonstrated that dietary intake affected age at natural menopause in a prospective cohort of British women. This study shows that high intakes of oily fish, fresh legumes as well as vitamin B6 and zinc are associated with a later onset of natural menopause while a high consumption of refined pasta and rice is associated with an earlier age at natural menopause.
