# Supplementary material for: Dietary intake and age at natural menopause: results from the UK Women’s Cohort Study
Source: J Epidemiol Community Health. 2018 Apr 30;72(8):733–40. doi: 10.1136/jech-2017-209887 (PMC6204950; doi:10.1136/jech-2017-209887)
Supplement: Supplementary file 1 [file jech-2017-209887supp001.pdf]

Online Supplementary material

Supplementary Table 1. Grouping of individual food items into 64 food groups

Food Group	Food Items
**Wholegrain products**	Crispbread, Brown bread & rolls, Wholemeal bread & rolls ,
**Refined grain products**	White bread & rolls, Chapattis, Nan, paratha, Papadums, Tortillas, Pitta Bread, Cream crackers, cheese biscuits
**Low fibre breakfast cereals**	Cream crackers, cheese biscuits, Sugar coated cereals, Non-sugar coated cereals
**High fibre breakfast cereals**	Porridge, Readybrek , Muesli, All bran, bran flakes, Weetabix, shredded wheat
**Plain Potatoes**	Potatoes, Jacket potato
**Potatoes with added fat**	Chips, Roast potatoes, Potato salad
**Refined pasta and rice**	White pasta, Macaroni cheese, White rice
**Wholegrain pasta and rice**	Wholemeal pasta, Brown rice, Wild rice
**Low fat dairy products**	Low fat yoghurt, Diet yoghurt, Dairy desserts, Low-fat cheese, Cottage cheese, Milk puddings, Half fat milk, Fat free milk
**High fat dairy products**	Thick & creamy yoghurt, Greek yoghurt, Fromage frais/Crème fraiche, Single/sour cream, Double/clotted cream, Ice cream, Cheese, Cheese and onion pastie, Whole milk, Channel island milk, Dried milk
**Butter and hard margarine**	Butter, Block margarine
**Margarine**	Other soft margarine, Polyunsaturated margarine, Monounsaturated margarine
**Low fat spreads**	Low fat spread, Very low fat spread
**High fat dressing**	Mayonnaise, French type dressing
**Low fat dressing**	Low calorie salad cream
**Soybean products**	Soya cheese, Soya yoghurt, Soy milk
**Textured vegetable protein**	Textured vegetable protein
**Pulses**	Lentils, dals, Chick peas, chanas, Hummus, Baked beans, Mung beans & red kidney beans, Black eyed beans, Butter beans/broad beans
**Eggs/eggs dishes**	Boiled/poached egg, Omelette, scrambled egg, Fried egg, Quiche
**Fish and fish dishes**	Fish fingers/cakes, Fried fish in batter, White fish, Fish pie/fish lasagne, Fish roe
**Oily fish**	Oily fish
**Shellfish**	Shellfish
**Red meat**	Beef, Beef stew, Pork, Pork stew/casserole, Lamb, Lamb stew/casserole, Meat – lasagne/moussaka/ravioli
**Poultry**	Chicken/turkey, Breadcrumbed, Chicken/turkey in creamy sauce, curry
**Processed meat**	Bacon, Beefburger/hamburger, Ham, Corned beef, Sausages, Meat pizza, Pies/pasties/sausage rolls, Liver pate/sausage, salami
**Offal**	Offal
**Vegetable dishes**	Quorn, Vegetarian chilli, Mixed bean casserole, Stir-fry vegetables, Vegetable – lasagne/moussaka/ravioli, Vegetable pate, Vegetable pizza
**Allium**	Leeks, Garlic
**Fresh legumes**	Peas, mushy peas, mange-tout, Green beans
**Mediterranean vegetables**	Sweetcorn, Courgettes, Olive, Aubergine, okra/ladies finger, Peppers
**Salad vegetables**	Avocado, Lettuce, Cucumber, Celery, Coleslaw, Low calorie coleslaw
**Cruciferous vegetables**	Broccoli, spring greens, kale, Cabbage, Cauliflower, Watercress, mustard & cress, Brussel sprouts
**Tomatoes**	Tomatoes – raw/canned/sauce
**Mushrooms**	Mushrooms
**Roots and tubers**	Carrots, Parsnips, Turnip, Swedes, Beetroot
**Stone fruits**	Peaches, Plum, Mangoes, Nectarines, Apricots
**Deep orange/yellow fruits**	Pineapple, Papaya, Melon
**Grapes**	Grapes
**Citrus family fruits**	Oranges, satsumas, grapefruit

**Rhubarb**	Rhubarb
**Berries**	Strawberries, Raspberries, Red currants/black currants, Kiwi fruit
**Bananas**	Bananas
**Pomes**	Apples, Pears
**Dried Fruits**	Dates, Figs, Prunes, Mixed dried fruits, Currants, raisins, sultanas
**Sauces**	Sauces
**Pickles/chutneys**	Tomato ketchup, Pickles/chutney/pesto sauce
**Soups**	Packet soups, Other-vegetable soups, Other-Meat soups, Low calorie soups
**Confectionery & spreads**	Fruit bars, Chocolate snack bars, Mini chocolate snack bars, Boiled sweets, toffees, mints, Chocolate/chocolate & nut spread, Jam/marmalade, Honey
**Nuts & Seeds**	Peanuts/Pistachio nuts, Cashew nuts & almonds, Pecan nuts/ Walnuts, Sunflower seeds/ sesame seeds, Nut Pâté, Peanut butter, Peanuts/pistachio nuts, Mixed nuts and raisins
**Savoury snacks**	Crisps, Other fried snacks, Low fat or baked snacks, Bombay mix
**Biscuits**	Plain biscuits, Chocolate biscuits, Sandwich/cream biscuits
**Cakes**	Fruitcake, Sponge cake
**Pastries and Puddings**	Buns/pastries, Scones/pancakes/muffins/crumpets, Fruit pies, Sponge puddings
**Tea**	Tea
**Herbal tea**	Herbal tea
**Coffee**	Coffee – instant/ground, Coffee – decaffeinated
**Other hot beverages**	Cocoa, Horlicks, Ovaltine, Low calorie hot chocolate
**Juices**	Orange juice, Other – pure juices
**Soft drinks**	Fruit squash, Fizzy soft drinks
**Low calorie/diet soft drinks**	Low calorie/diet soft drinks
**Wines**	Wines
**Beer and cider**	Beer, Cider
**Port, sherry, liqueurs**	Port, sherry, liqueurs
**Spirits**	Spirits

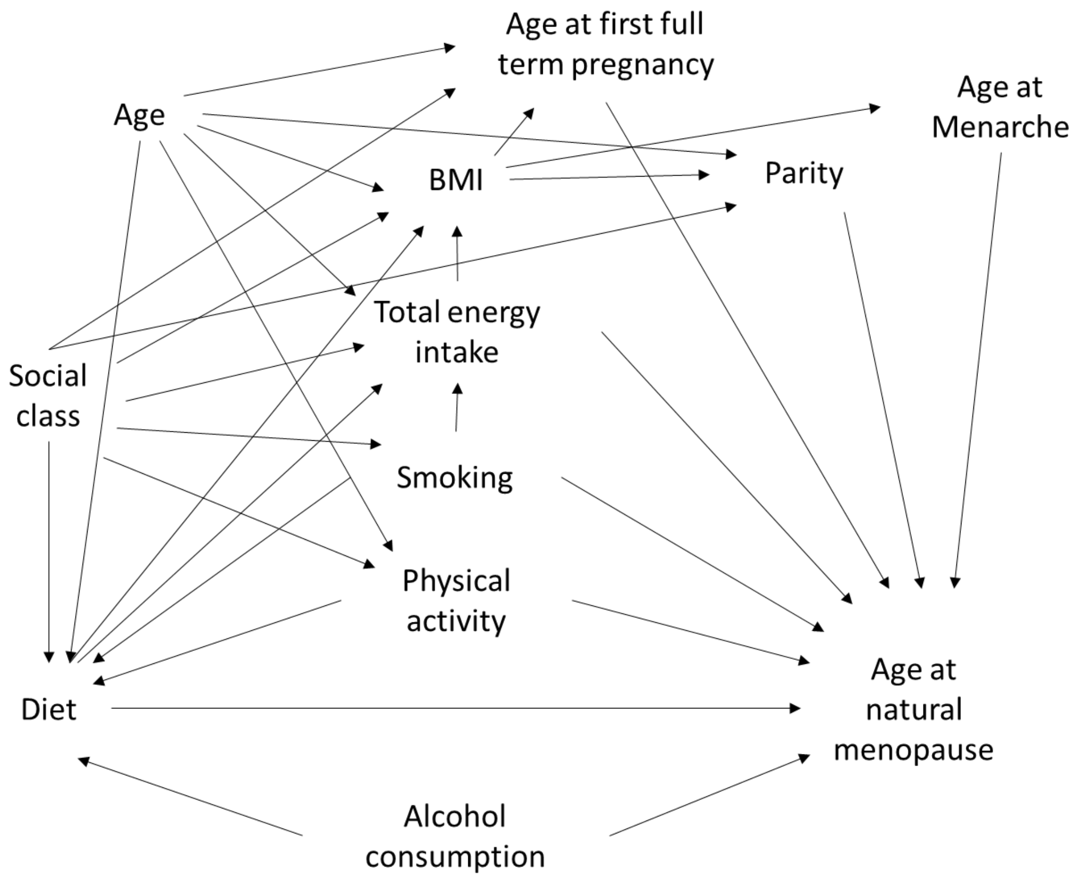

Supplementary Figure 1. Directed acrylic graph – for assessment of covariates

Supplementary Table 2. Sensitivity analysis of daily food groups intake/portion size and age at natural menopause by vegetarian status, fully adjusted model

	Vegetarians (n=323)			Non-vegetarians (n=501)		
Daily intake/ portion size	Difference in age at natural menopause (y)	99% CI	*P* value	Difference in age at natural menopause (y)	99% CI	*P* value
Starchy foods						
Wholegrain products/ 33g	0.0	-0.2 to 0.3	0.589	0.1	-0.0 to 0.3	0.219
Refined grain products/ 51g	-0.1	-0.7 to 0.5	0.665	-0.2	-0.7 to 0.3	0.300
Low fibre breakfast cereals/ 40g	0.0	-1.7 to 1.8	0.945	-0.1	-1.3 to 1.1	0.829
High fibre breakfast cereals/ 85g	0.1	-0.6 to 0.8	0.755	0.4	-0.2 to 1.0	0.108
Plain Potatoes/ 210g	0.5	-0.6 to 1.6	0.260	0.6	-0.4 to 1.6	0.140
Potatoes with added fat/ 127g	-0.2	-3.0 to 2.6	0.828	-0.3	-2.3 to 1.6	0.651
Refined pasta and rice/ 210g	-1.4	-3.3 to 0.5	0.057	-1.6	-3.4 to 0.3	0.031
Wholegrain pasta and rice/ 197 g	0.9	-1.1 to 2.8	0.237	0.9	-1.2 to 3.0	0.256
Protein and fat food sources						
Low fat dairy products/ 80g	-0.1	-0.2 to 0.1	0.171	0.1	-0.0 to 0.2	0.228
High fat dairy products/ 75g	0.0	-0.2 to 0.3	0.656	-0.2	-0.4 to 0.1	0.074
Butter and hard margarine/ 10g	0.0	-0.6 to 0.5	0.852	0.3	-0.2 to 0.7	0.103
Margarine/ 9g	-0.1	-0.5 to 0.3	0.379	-0.1	-0.5 to 0.3	0.675
Low fat spreads/ 7g	0.2	-0.4 to 0.8	0.320	0.0	-0.4 to 0.3	0.749
High fat dressing/ 23g	0.0	-1.5 to 1.5	0.979	0.1	-1.4 to 1.6	0.870
Low fat dressing/ 30g	0.9	-2.5 to 4.3	0.475	1.2	-1.5 to 3.9	0.262
Soybean products/ 62g	0.0	-0.1 to 0.2	0.436	-0.1	-0.4 to 0.3	0.630
Textured vegetable protein/ 130g	1.1	-8.8 to 10.9	0.782	-9.9	-43.0 to 23.2	0.440
Pulses/ 91g	0.1	-0.9 to 1.0	0.885	-0.2	-1.3 to 0.9	0.653
Eggs/eggs dishes/ 88g	0.1	-1.8 to 2.1	0.865	1.1	-1.0 to 3.1	0.187
Fish and fish dishes/ 140g	-1.5	-4.7 to 1.7	0.227	1.8	-1.3 to 5.0	0.132
Oily fish/ 90g	0.7	-4.0 to 5.3	0.713	3.4	0.2 to 6.5	0.005
Shell fish/ 60g	-1.8	-12.7 to 9.0	0.663	2.3	-5.9 to 10.4	0.476
Red meat/ 189g	12.5	-136.4 to 161.4	0.828	0.7	-1.5 to 2.9	0.400
Processed meat/ 74g	-4.2	-23.1 to 14.7	0.566	-0.3	-2.5 to 2.0	0.743
Poultry/ 143g	7.4	-3.1 to 17.8	0.069	-0.6	-3.4 to 2.3	0.607
Offal/ 100g	.	.	.	1.8	-8.5 to 12.1	0.647
Vegetables						
Vegetable dishes/ 214g	0.0	-1.2 to 1.1	0.924	-0.3	-1.7 to 1.1	0.593

Allium/ 39g	0.6	-0.7 to 1.9	0.219	0.5	-0.8 to 1.8	0.282
Fresh legumes/ 75g	0.3	-1.0 to 1.5	0.556	1.4	0.2 to 2.7	0.003
Mediterranean vegetables/ 60g	0.0	-0.8 to 0.8	0.904	0.4	-0.6 to 1.3	0.314
Salad vegetables/ 43g	0.3	-0.3 to 1.0	0.210	0.7	0.0 to 1.3	0.009
Cruciferous vegetables/ 75g	0.3	-0.2 to 0.8	0.143	0.4	-0.1 to 0.9	0.051
Tomatoes/ 83g	-0.3	-1.1 to 0.5	0.313	0.8	-0.2 to 1.8	0.043
Mushrooms/ 34g	0.1	-1.5 to 1.8	0.831	0.5	-1.2 to 2.2	0.424
Roots and tubers/ 66g	0.2	-0.5 to 0.9	0.550	0.8	-0.1 to 1.7	0.016
Fruits						
Stone fruits/ 49g	0.2	-0.7 to 1.1	0.604	1.0	0.5 to 2.5	0.083
Deep orange & yellow fruits/ 118g	0.5	-0.4 to 1.4	0.139	0.7	-0.6 to 2.0	0.189
Grapes/ 100g	0.6	-0.5 to 1.7	0.175	0.9	-0.6 to 2.3	0.117
Citrus family fruits/ 92g	0.3	-0.5 to 1.1	0.363	0.1	-0.7 to 1.0	0.651
Rhubarb/ 130g	-0.1	-2.0 to 1.8	0.862	1.5	-0.5 to 3.5	0.056
Berries/ 48g	0.2	-0.5 to 0.8	0.514	0.5	-0.4 to 1.3	0.157
Bananas/ 100g	0.0	-0.8 to 0.8	0.980	0.1	-0.6 to 0.9	0.662
Pomes/ 116g	0.0	-0.5 to 0.5	0.865	0.1	-0.3 to 0.6	0.451
Dried Fruits/ 28g	0.2	-0.5 to 0.9	0.371	0.5	-0.1 to 1.2	0.025
Other food groups						
Sauces/ 83g	0.1	-3.6 to 3.8	0.936	0.2	-3.1 to 3.5	0.888
Pickles/Chutneys/ 35g	-0.6	-2.4 to 1.3	0.429	0.2	-1.7 to 2.1	0.827
Soups/ 163g	1.3	-0.5 to 3.2	0.065	0.7	-0.7 to 2.1	0.222
Confectionary & spreads/ 44g	-0.1	-0.6 to 0.3	0.455	0.1	-0.4 to 0.6	0.690
Nuts and seeds/ 24g	0.2	-0.26 to 0.6	0.279	0.2	-0.5 to 0.9	0.476
Savoury snacks/ 26g	0.1	-1.2 to 1.4	0.863	-1.7	-3.1 to -0.4	0.001
Biscuits/ 15g	-0.1	-0.7 to 0.5	0.567	-0.3	-0.7 to 0.2	0.130
Cakes/ 66g	-1.2	-3.4 to 1.1	0.183	0.7	-1.4 to 2.8	0.389
Pastries and Puddings/ 84g	-0.1	-1.7 to 1.5	0.912	-0.8	-2.3 to 0.8	0.190
Drinks and beverages						
Tea/ 260g	-0.1	-0.3 to 0.2	0.567	-0.2	-0.4 to 0.0	0.045
Herbal tea/ 260g	0.1	-0.4 to 5.8	0.533	0.3	-0.2 to 0.7	0.151
Coffee/ 190g	0.0	-0.3 to 0.2	0.767	0.1	-0.1 to 0.3	0.162
Other hot beverages/ 23g	0.0	-0.6 to 0.6	0.975	0.4	-0.5 to 1.2	0.272
Juices/ 145g	-0.2	-0.9 to 0.5	0.489	0.3	-0.3 to 0.8	0.219
Soft drinks/ 111g	-0.2	-1.4 to 0.1	0.674	-1.3	-2.5 to -0.2	0.003

Low calorie/diet soft drinks/ 161g	0.1	-0.8 to 0.9	0.812	-0.4	-1.3 to 0.6	0.301
Wines/ 1g	0.4	-0.6 to 1.3	0.360	0.0	-0.9 to 0.8	0.882
Beer and cider/ 1g	-0.6	-1.8 to 0.6	0.191	-0.3	-1.4 to 0.8	0.449
Port, sherry, liqueurs/ 1g	1.6	-1.0 to 4.3	0.112	0.7	-1.2 to 2.7	0.346
Spirits/ 1g	0.1	-1.1 to 1.2	0.887	-0.4	-1.6 to 0.7	0.345

Supplementary Table 3. Sensitivity analysis of daily food groups intake/portion size and age at natural menopause by parity, fully adjusted model

	**Nulliparous (n=179)**			**Multiparous (n=645)**		
Daily intake/ portion size	Difference in age at natural menopause (y)	99% CI	*P* value	Difference in age at natural menopause (y)	99% CI	*P* value
Starchy foods						
Wholegrain products/ 33g	0.0	-0.3 to 0.3	0.899	0.1	-0.1 to 0.2	0.344
Refined grain products/ 51g	0.0	-0.9 to 0.9	0.939	-0.3	-0.7 to 0.2	0.119
Low fibre breakfast cereals/ 40g	-0.4	-2.8 to 2.0	0.643	-0.1	-1.3 to 1.0	0.769
High fibre breakfast cereals/ 85g	0.1	-0.9 to 1.0	0.890	0.2	-0.4 to 0.7	0.366
Plain Potatoes/ 210g	0.4	-1.0 to 1.7	0.501	0.4	-0.5 to 1.3	0.202
Potatoes with added fat/ 127g	0.1	-3.9 to 4.1	0.948	-0.3	-2.1 to 1.4	0.637
Refined pasta and rice/ 210g	-2.0	-5.6 to 1.6	0.157	-1.9	-3.3 to -0.4	0.001
Wholegrain pasta and rice/ 197 g	1.0	-2.0 to 4.0	0.388	0.5	-1.1 to 2.1	0.448
Protein and fat food sources						
Low fat dairy products/ 80g	0.0	-0.3 to 0.2	0.575	0.0	-0.1 to 0.1	0.722
High fat dairy products/ 75g	0.1	-0.3 to 0.5	0.654	-0.1	-0.3 to 0.1	0.227
Butter and hard margarine/ 10g	0.0	-0.7 to 0.6	0.862	0.2	-0.2 to 0.6	0.120
Margarine/ 9g	-0.1	-0.7 to 0.5	0.677	-0.2	-0.6 to 0.1	0.061
Low fat spreads/ 7g	0.2	-0.8 to 1.2	0.682	0.0	-0.3 to 0.4	0.969
High fat dressing/ 23g	-0.1	-2.1 to 1.8	0.862	-0.1	-1.4 to 1.2	0.830
Low fat dressing/ 30g	-0.2	-4.4 to 4.0	0.900	1.1	-1.4 to 3.5	0.266
Soybean products/ 62g	0.0	-0.3 to 0.2	0.690	0.0	-0.2 to 0.2	0.900
Textured vegetable protein/ 130g	-12.6	-39.7 to 14.6	0.230	-3.1	-12.8 to 6.5	0.400
Pulses/ 91g	-0.6	-1.9 to 0.8	0.290	-0.3	-1.1 to 0.5	0.298
Eggs/eggs dishes/ 88g	1.8	-1.5 to 5.1	0.167	0.1	-1.5 to 1.7	0.829
Fish and fish dishes/ 140g	3.9	-1.5 to 9.3	0.064	0.4	-1.9 to 2.7	0.628
Oily fish/ 90g	3.2	-1.0 to 7.4	0.046	3.3	0.3 to 6.3	0.005
Shell fish/ 60g	7.6	-10.0 to 25.1	0.262	0.5	-6.4 to 7.4	0.847
Red meat/ 189g	3.0	-1.3 to 7.3	0.073	1.1	-0.8 to 3.0	0.150
Processed meat/ 74g	1.6	-2.8 to 6.1	0.341	0.6	-1.4 to 2.5	0.435
Poultry/ 143g	5.2	0.1 to 10.3	0.008	0.2	-2.4 to 2.7	0.869
Offal/ 100g	13.3	-10.5 to 37.0	0.148	4.7	-5.6 to 14.9	0.242
Vegetables						
Vegetable dishes/ 214g	-1.4	-3.1 to 0.4	0.047	-0.4	-1.4 to 0.5	0.236

Allium/ 39g	-0.1	-2.0 to 1.9	0.942	0.6	-0.5 to 1.7	0.135
Fresh legumes/ 75g	-0.1	-1.9 to 1.7	0.872	1.1	0.1 to 2.1	0.006
Mediterranean vegetables/ 60g	-0.3	-1.5 to 0.9	0.501	0.0	-0.7 to 0.7	0.938
Salad vegetables/ 43g	0.2	-0.7 to 1.1	0.522	0.4	-0.1 to 1.0	0.032
Cruciferous vegetables/ 75g	0.1	-0.9 to 1.0	0.874	0.4	-0.1 to 0.8	0.023
Tomatoes/ 83g	-0.4	-1.7 to 0.8	0.351	0.2	-0.5 to 0.9	0.507
Mushrooms/ 34g	-1.4	-4.5 to 1.7	0.234	0.4	-0.9 to 1.7	0.471
Roots and tubers/ 66g	0.3	-1.0 to 1.6	0.567	0.4	-0.2 to 1.1	0.086
Fruits						
Stone fruits/ 49g	0.4	-2.0 to 2.7	0.695	0.3	-0.5 to 1.1	0.316
Deep orange & yellow fruits/ 118g	0.0	-2.0 to 1.9	0.978	0.6	-0.2 to 1.4	0.057
Grapes/ 100g	2.5	0.1 to 4.9	0.008	0.4	-0.5 to 1.4	0.248
Citrus family fruits/ 92g	0.0	-1.1 to 1.1	0.976	0.2	-0.5 to 0.9	0.481
Rhubarb/ 130g	1.0	-2.6 to 4.5	0.475	0.7	-0.9 to 2.2	0.256
Berries/ 48g	0.4	-1.2 to 1.9	0.538	0.2	-0.4 to 0.7	0.404
Bananas/ 100g	0.0	-1.0 to 1.0	0.989	0.0	-0.6 to 0.7	0.890
Pomes/ 116g	0.2	-0.5 to 0.9	0.456	0.0	-0.4 to 0.4	0.856
Dried Fruits/ 28g	0.2	-0.7 to 1.2	0.478	0.4	-0.1 to 1.0	0.029
Other food groups						
Sauces/ 83g	-0.9	-6.3 to 4.5	0.673	0.1	-2.7 to 2.8	0.963
Pickles/Chutneys/ 35g	-1.0	-3.6 to 1.6	0.324	-0.1	-1.6 to 1.4	0.869
Soups/ 163g	1.0	-1.6 to 3.5	0.316	0.8	-0.5 to 2.1	0.108
Confectionary & spreads/ 44g	0.1	-0.5 to 0.7	0.684	-0.1	-0.4 to 0.3	0.716
Nuts and seeds/ 24g	0.2	-0.9 to 1.3	0.580	0.1	-0.3 to 0.5	0.499
Savoury snacks/ 26g	0.0	-2.1 to 2.2	0.964	-1.1	-2.1 to -0.0	0.009
Biscuits/ 15g	-0.2	-0.9 to 0.5	0.491	-0.2	-0.6 to 0.2	0.141
Cakes/ 66g	-0.7	-3.5 to 2.2	0.533	0.2	-1.7 to 2.0	0.793
Pastries and Puddings/ 84g	-0.5	-3.7 to 2.6	0.650	-0.5	-1.7 to 0.7	0.271
Drinks and beverages						
Tea/ 260g	-0.2	-0.5 to 0.1	0.052	-0.1	-0.3 to 0.1	0.161
Herbal tea/ 260g	0.4	-0.2 to 0.9	0.066	0.0	-0.4 to 0.4	0.868
Coffee/ 190g	0.0	-0.4 to 0.3	0.800	0.1	-0.1 to 0.3	0.214
Other hot beverages/ 23g	-0.3	-1.4 to 0.7	0.393	0.3	-0.3 to 0.9	0.145
Juices/ 145g	0.0	-1.0 to 1.0	0.992	0.1	-0.4 to 0.6	0.522
Soft drinks/ 111g	-1.2	-2.7 to 0.3	0.032	-0.6	-1.6 to 0.4	0.098

Low calorie/diet soft drinks/ 161g	-0.6	-2.1 to 0.9	0.273	-0.1	-0.8 to 0.6	0.759
Wines/ 1g	0.7	-0.2 to 1.7	0.033	-0.3	-1.2 to 0.5	0.332
Beer and cider/ 1g	-0.5	-1.6 to 0.6	0.274	-0.4	-1.5 to 0.6	0.277
Port, sherry, liqueurs/ 1g	2.3	-0.8 to 5.3	0.052	0.6	-1.3 to 2.4	0.408
Spirits/ 1g	0.3	-1.8 to 2.4	0.722	-0.3	-1.2 to 0.6	0.399

Supplementary Table 4. Sensitivity analysis of daily food groups intake/portion size and age at natural menopause by presence of diabetes at baseline, fully adjusted model

Daily intake/ portion size	Difference in age at natural menopause[a]	99% CI	*P* value
Starchy food sources			
Wholegrain products/ 33g	0.1	-0.1 to 0.2	0.272
Refined grain products/ 51g	-0.1	-0.5 to 0.2	0.347
Low fibre breakfast cereals/ 40g	0.0	-1.0 to 1.0	0.944
High fibre breakfast cereals/ 85g	0.2	-0.3 to 0.6	0.338
Plain Potatoes/ 210g	0.6	-0.2 to 1.3	0.046
Potatoes with added fat/ 127g	-0.1	-1.7 to 1.6	0.923
Refined pasta and rice/ 210g	-1.6	-2.9 to -0.2	0.003
Wholegrain pasta and rice/ 197 g	0.4	-1.0 to 1.8	0.483
Protein and fat food sources			
Low fat dairy products/ 80g	0.0	-0.1 to 0.1	0.642
High fat dairy products/ 75g	-0.1	-0.3 to 0.1	0.212
Butter and hard margarine/ 10g	0.1	0.2 to 0.5	0.283
Margarine/ 9g	-0.1	-0.4 to 0.1	0.196
Low fat spreads/ 7g	0.1	-0.2 to 0.4	0.320
High fat dressing/ 23g	-0.1	-1.2 to 0.9	0.724
Low fat dressing/ 30g	1.0	-1.2 to 3.2	0.255
Soybean products/ 62g	0.0	-0.2 to 0.1	0.791
Textured vegetable protein/ 130g	-4.2	-13.2 to 4.7	0.225
Pulses/ 91g	-0.3	-1.0 to 0.3	0.192
Eggs/eggs dishes/ 88g	0.5	-0.9 to 2.0	0.338
Fish and fish dishes/ 140g	1.2	-0.9 to 3.3	0.148
Oily fish/ 90g	3.0	0.5 to 5.5	0.002
Shell fish/ 60g	0.6	6.0 to 7.2	0.818
Red meat/ 189g	1.3	-0.4 to 3.1	0.044
Processed meat/ 74g	1.2	-0.6 to 3.0	0.093
Poultry/ 143g	1.9	-0.6 to 4.4	0.047
Offal/ 100g	6.6	-2.8 to 16.0	0.071
Vegetables			
Vegetable dishes/ 214g	-0.6	-1.4 to 0.2	0.054
Allium/ 39g	0.5	0.5 to 1.4	0.196

Fresh legumes/ 75g	0.9	0.0 to 1.8	0.007
Mediterranean vegetables/ 60g	0.0	-0.6 to 0.6	0.991
Salad vegetables/ 43g	0.3	-0.1 to 0.8	0.057
Cruciferous vegetables/ 75g	0.3	-0.1 to 0.6	0.047
Tomatoes/ 83g	0.0	-0.6 to 0.6	0.985
Mushrooms/ 34g	0.2	-1.0 to 1.4	0.672
Roots and tubers/ 66g	0.3	-0.2 to 0.9	0.111
Fruits			
Stone fruits/ 49g	0.2	-0.5 to 1.0	0.425
Deep orange & yellow fruits/ 118g	0.5	-0.2 to 1.2	0.088
Grapes/ 100g	0.6	-0.2 to 1.5	0.065
Citrus family fruits/ 92g	0.2	-0.4 to 0.7	0.443
Rhubarb/ 130g	0.8	-0.6 to 2.1	0.149
Berries/ 48g	0.2	-0.3 to 0.7	0.293
Bananas/ 100g	0.1	-0.5 to 0.6	0.736
Pomes/ 116g	0.0	-0.3 to 0.4	0.846
Dried Fruits/ 28g	0.3	-0.1 to 0.8	0.051
Other food groups			
Sauces/ 83g	-0.2	-2.7 to 2.2	0.831
Pickles/Chutneys/ 35g	-0.3	-1.6 to 1.1	0.622
Soups/ 163g	0.7	-0.4 to 1.9	0.096
Confectionary & spreads/ 44g	0.0	-0.3 to 0.3	0.889
Nuts and seeds/ 24g	0.1	-0.3 to 0.4	0.643
Savoury snacks/ 26g	-0.8	-1.8 to 0.1	0.020
Biscuits/ 15g	-0.2	-0.5 to 0.2	0.214
Cakes/ 66g	-0.3	-2.0 to 1.2	0.569
Pastries and Puddings/ 84g	-0.5	-1.6 to 0.6	0.263
Drinks and beverages			
Tea/ 260g	-0.1	-0.3 to 0.1	0.100
Herbal tea/ 260g	0.1	-0.2 to 0.5	0.243
Coffee/ 190g	0.1	-0.1 to 0.2	0.301
Other hot beverages/ 23g	0.1	-0.4 to 0.6	0.763
Juices/ 145g	0.1	-0.3 to 0.5	0.547
Soft drinks/ 111g	-0.8	-1.6 to 0.0	0.015
Low calorie/diet soft drinks/ 161g	-0.1	-0.7 to 0.5	0.760

Wines/ 1g	0.1	-0.5 to 0.8	0.573
Beer and cider/ 1g	-0.4	-1.2 to 0.3	0.126
Port, sherry, liqueurs/ 1g	1.0	-0.5 to 2.6	0.086
Spirits/ 1g	-0.1	-1.0 to 0.7	0.673

[a] Model adjusted for the following factors: Physical activity level, alcohol consumption, smoking, social class, presence of diabetes (n=801)
